# Book Reviews

**Published:** 1915-02

**Authors:** 


### BOOK REVIEWS

Books mentioned may be inspected at and ordered through this office. So far as possible, books received in any month will be reviewed in the issue of the second month following.

**Text Book For Midwives.** John S. Fairbairn, M. A., B. M., B. Ch., F. R. C. P., F. R. C. S., London. Oxford University Press, American Branch, 35 West 32 street, N. Y. 316 pages 3 plates and 104 illustrations, $3.75.

An ingenious method to facilitate reference is the superposition of transparent paper with names of parts, over the large cuts. The illustrations generally are of high order. The only adverse criticism that can be made is that practically no American midwives will or can use so elaborate a text book. Here is the difference between the status of midwives in Europe and the States: in Europe, obstetric practice by midwives is recognized as normal and every endeavor is made to regulate their practice and to educate them. In this country, midwives are tolerated in deference to the customs of the more ignorant portion of the population, an effort is made to secure reasonable degrees of practical efficiency in simple cases but it has not been found practicable to demand a thorough training and many hold that, to go beyond the license to attend the simplest labors and to attempt a higher standard of training would result in more harm than good by giving the selfassurance that goes with imperfect knowledge. So far as we can judge, this book is well adapted to the use of students and practitioners of medicine. It is far beyond the brief manual or compend although it does not pretend to conform to the requirements of an elaborate, specialized monograph.

---

**Child Training as an Exact Science.** Geo. W. Jacoby, M. D., N. Y. 384 pages, 15 full-page illustrations, $1.50, Funk & Wagnalls Co., N. Y.

This is an application of psychology and devotes a good deal of attention to the modern methods of measuring intellectual development, although physical defects receive their due share of discussion. The book is free from sensationalism and radical attempts at novelty and it might well be prescribed by the family physician for all intelligent parents to follow. Without wishing to appear captious, we are inclined to question whether child training is so much an exact science as it is a consummate art. At any rate, we venture the opinion that the value of this book lies not so much with the scientific accomplishments of students of child psychology as with the practical experience and sound sense which the author has shown in adapting these scientific foundations to the building of character and physical health.

---

**Diseases of The Stomach.** Charles G. Stockton, M. D., Buffalo. D. Appleton & Co., N. Y. and London, 774 pages, 5 plates, 22 radiograms and 65 illustrations in text.

We wish, at the outset, to express our appreciation of the personal inscription in the copy sent us and of the friendly letter accompanying the book. It is always a pleasure to note the authorship of a professional colleague in our own locality, of a friend, of a teacher and in this case, all of these items are combined. The work is dedicated to the author's father and first teacher in medicine, the late Dr. Charles Lewis Stockton. Credit is given in the preface to several collaborators along different lines of study.

From a comprehensives treatise of large volume, such as the present, it is difficult to make extracts to show adequately the excellence of the matter but we venture to do so, at random. From the wording of the discussion of auscultation of the stomach: "Meltzer speaks of primary and secondary sounds . . ." we infer that the author's experience has been like our own and that the exact classification of deglutition murmurs has not proved feasible. In speaking of the size and shape of the stomach, the lack of agreement among anatomists, clinicians and radiographers is frankly noted—but we think a trifle too modestly from the clinician's standpoint, since immediate comparison of auscultatory percussion findings with post mortem section actually does show the reliability of the clinician's land marks. In another place, detailed consideration is given to the interpretation of X-Ray pictures—and it should be noted that the word interpretation is highly significant for this method is not simply a matter of inspection one step removed from what was formerly so called. The statistic study of cancer, included in a clinical text book, is of the utmost value in the present state of our knowledge of this disease. While the author draws largely from his own experience, he has included the work of various other investigators and even special tests regarding which a non-committal attitude is still necessary. Very wisely, we think, Dr. Stockton has avoided the pitfall of combining dietetics and gastrology. While general principles of alimentation have been clearly stated and special dietetic methods of treatment have received due attention, no attempt has been made to divert energy from the subject chosen and to invade the wide field of systematic dietetics. Especially valuable are the discussions of gastric lesions and reactions in general diseases, as tuberculosis, syphilis, etc., and those in which the heart, blood vessels, kidneys and liver modify the physiology of the stomach through the circulation by inducing chemic disturbances and otherwise.

It is almost unnecessary to conclude with a hearty recommendation of this book to specialist and general practitioner, not forgetting to include in the former category, the surgeon and the radiologist.

---

**International Clinics,** Vol. 4, 24th annual series, 1914. Edited by Henry W. Cattell, A. M., M. D., Philadelphia, published by the J. B. Lippincott Co., 314 pages, with photographic and colored plates.

Having been connected with this publication from its inception, as a correspondent or rather as a reporter, if so humble a term were ever used in medical journalism, and later at various times as a contributor, we feel some degree of personal interest in noting the long continuance, on a high grade, of this clinical review of the various branches of medicine. Western N. Y. is represented by an extensive monograph on the Auscultatory Method of Blood Pressure Determination, by Dr. John M. Swan, of Rochester, and by a case report (peritoneal adhesions masking ascites) by the editor of this journal.

---

**Selected Addresses.** James Tyson, M. D., LL. D., Philadelphia. P. Blakiston's Son & Co., Philadelphia, 366 pages, $1.75.

These addresses are of a historic, biographic and commemorative nature and mark the highest attainment of medical literature off the beaten path of technicality.

---

**Fever—Its Thermataxis and Metabolism.** Isaac Ott, A. M., M. D., Philadelphia, published by Paul B. Hoeber, 67-69 East 59th street, N. Y. 166 pages, illustrated, $1.50.

This is a monograph divided into three lectures, presenting in a logical sequence the physiologic data and clinical applications of an important and neglected subject. The evidence is presented, with historic references, succinctly and briefly but otherwise with almost legal accuracy. Two thermogenic centers are stated: the tuber cinereum and the corpus striatum; and two inhibitory centers: the cruciate and the sylvian. Three methods of study are possible, thermometry, calorimetry, and indirect calorimetry by observation of respirotary exchange. The author is both a physiologist and a clinician, hence we have the ideal comparison of animal experiment and clinical experience, as well as an ideally direct comparison of scientific principles with practical deductions, as to diagnosis, therapeutics and, especially diet. Every physician should read this book carefully. It will give him new ideas and explode some old notions.

---

**Carcinoma of the Thyroid in the Salmonoid Fishes.** Harvey R. Gaylord and Millard C. Marsh, with collaboration of Frederick C. Busch and Burton T. Simpson. Published by the State Institute for the Study of Malignant Disease, Buffalo, printed at the Government Printing Office, Washington. Serial No. 99. 524 pages of text, 110 plates.

This is a work of seven years, of the highest technical nature, carried on with the best equipment. On the one hand, it leads to economic conservation of an important natural food supply, on the other to practical, humanitarian results both in regard to cancer and to thyroid disease of other nature. The work is obviously so technical and so detailed that it does not readily lend itself to a review of the ordinary kind nor will many medical men have the patience to read it in full. It is rather a mine of information for experts and a foundation for future study along one of the lines indicated. As the establishment and maintenance of such an institution as that under whose auspices this work has been carried out, is often subject to legislative criticism on utilitarian grounds, it may be well to emphasize the value of professional influence to insure the continuance of state support and of private philanthropy directed toward the maintenance of research institutions. Just as patients with a disease of twenty years' standing sometimes demand a cure in a week, there has been a disposition to demand that a disease, which has baffled human efforts for the whole period of human history, should be rendered absolutely diagnosticable and curable, in return for a few years of state support. The necessity of deep and broad research has not been appreciated. A report of this kind may well teach a lesson. It is altogether probable that, from the strictly ultilitarian standpoint, the information contained in this publication, applied to pisciculture, will save the state—without regard to the value to other states and countries—every year, far more than the maximum expense of the entire institution. Yet, from the standpoint of the institution, this information was obtained in the endeavor to follow out every line of study which could have a bearing on the comprehension of malignant disease. And such lines of study have probably been most of all subjected to the criticism that they were outside the purposes of the institution.

---

**Gonorrhoea and Its Complications in the Male and Female.** David Watson, M. B., C. M., Glasgow. Published by Paul B, Hoeber, 69 E. 59, N. Y. 375 pages, 72 illustrations, and 12 plates, 9 colored. $3.75.

Unfortunately, many of the medical books by British authors received for review, are too brief and superficial to have much real value to the thorough student. It is refreshing, therefore, to note at the outset that the present author has properly appreciated the magnitude of his task. However, it would be unjust even to imply that the sole merit of this book is its size, or that the author has failed to appreciate the value of brevity. Indeed, the book is brief in the true sense of avoiding prolixity of discussion. For example, on a single page, is presented in tabular form, a list of practically all of the chemicals used for urethral injections, 45 in number, with statements of strength of solution for ordinary injection, instillation and irrigation. The subject is well covered, both in its scientific aspects and as regards practical diagnosis and therapeutics. What may be termed the sociologic aspects of gonorrhoea are adequately treated but without the unnecessary moralizing and discussion of details which detract from the technical value of some books.

---

**Mechano-Therapeutics in General Practice.** G. de Swietochowski, M. D., M. R. C. S., London. Published by Paul B. Hoeber, 69 E. 59, N. Y. 141 pages, 31 illustrations. $1.50.

After a very brief consideration of general principles, including indications and contra-indications, the detailed application of mechanic methods including various forms of massage, is considered under the general headings of Surgical,

Medical and Special. Fractures, almost bone by bone, sprains and dislocations, deformities and various injuries to soft parts and chronic inflammatory conditions of joints and soft parts are included under the first category. The circulatory, respiratory, digestive, urinary, nervous systems and constitutional diseases such as rickets, gout, adiposity, anaemia, etc., and the lesions and disturbances of the nervous system are considered seriatim. Gynaecology, obstetrics, rhinelaryngology, ophthalmology, dermatology and odontology are also available fields. The various forms of manipulation, including tapotement, kneading, stroking, massage and use of exercises are described as they are introduced into the special considerations and are illustrated.

This is a valuable work for the general practitioner and is especially adapted to his use by ease of reference.

---

**Therapeutics of the Circulation.** Sir Lauder Brunton, M. D., D. Cc., L.L.D., F. R. C. P., F. R. S., London. Published by Paul B. Hoeber, 69 E. 59, N. Y. 2d edition, 536 pages. $2.50.

It is important to note that this book is not confined to the discussion of blood pressure nor to the description of elaborate instruments, though including the modern methods of study of the circulation. It is a general discussion, of thorough nature, of the whole subject as designated and while giving due place to strictly physiologic principles, it is eminently practical. The author does not disdain the use of drugs. He is critical, by no means credulous, but far from therapeutic nihilism. He discusses the heart and vessels and their relation to other organs in a broad way but always with a view to focussing on the needs of the actual practitioner.

---

**Men of 1914.** Published by the Men of Nineteen-Fourteen Co., Chicago. 846 pages. $10.

This is a biographic index of living prominent men of all vocations who are considered Builders of the Nation. While it is difficult always to make the selections properly inclusive and exclusive, the compilers have solved these problems to the best of their ability and have confined themselves to ordinary biographic data and statements of what the men do and what they are interested in without attempting eulogies which must have been mainly autobiographic. It is a useful reference work, attractively printed and bound.

---

**Secrets of the German War Office.** Dr. Armgaard Karl Graves, published by McBride, Nast & Co.

This is an extremely interesting and instructive book. The author does not reveal his true identity. We are not quite certain whether the book really is an autobiographic account of experiences as a diplomatic spy or whether, with some recollections of travel, a file of the Literary Digest, and recent visits to the movies to see "My Official Wife" and "The Typhoon," the story was composed in New York. In either case, it is worth reading and it divulges nothing which would particularly embarrass the German cause.

---

**Social Forces in England and America.** H. G. Wells.

We wish merely to call attention to the chapter on Doctors, a curious combination of good ideals and ignorance as to the actual realization of these ideals or of the practical objections to the realization of some of them.

